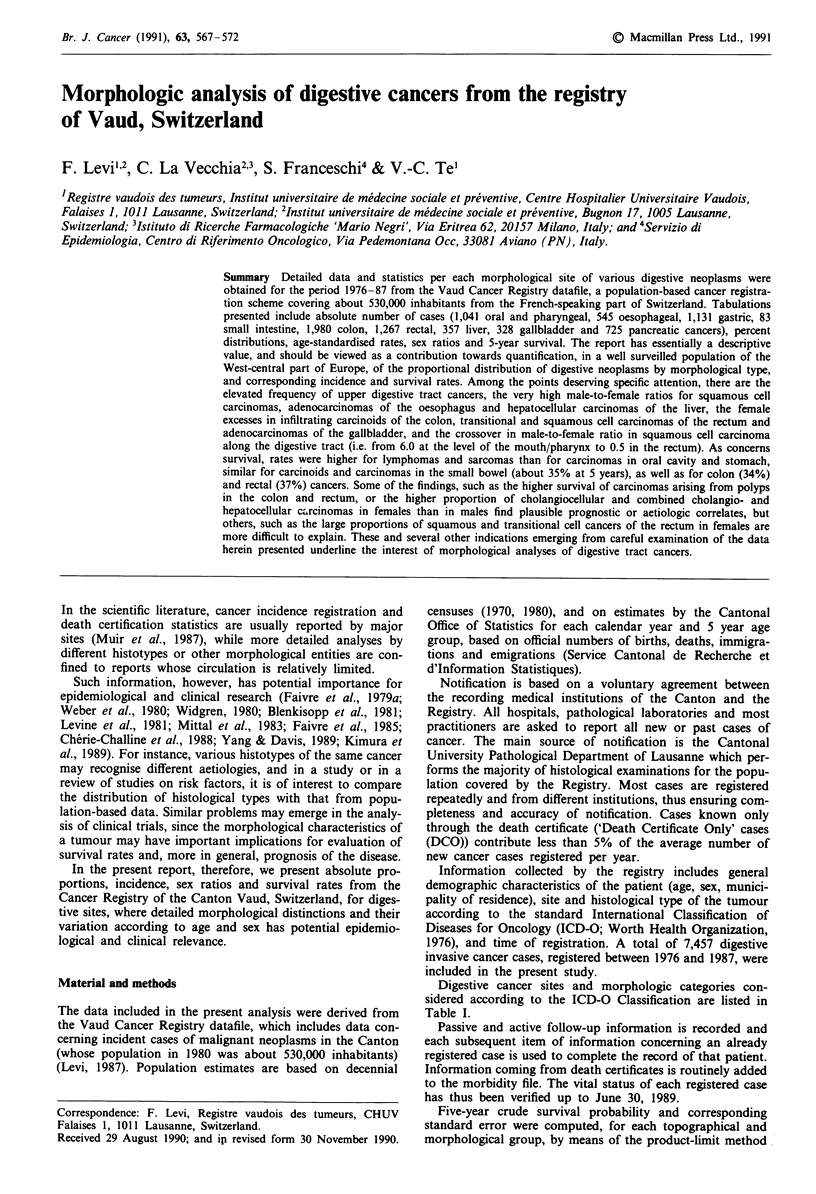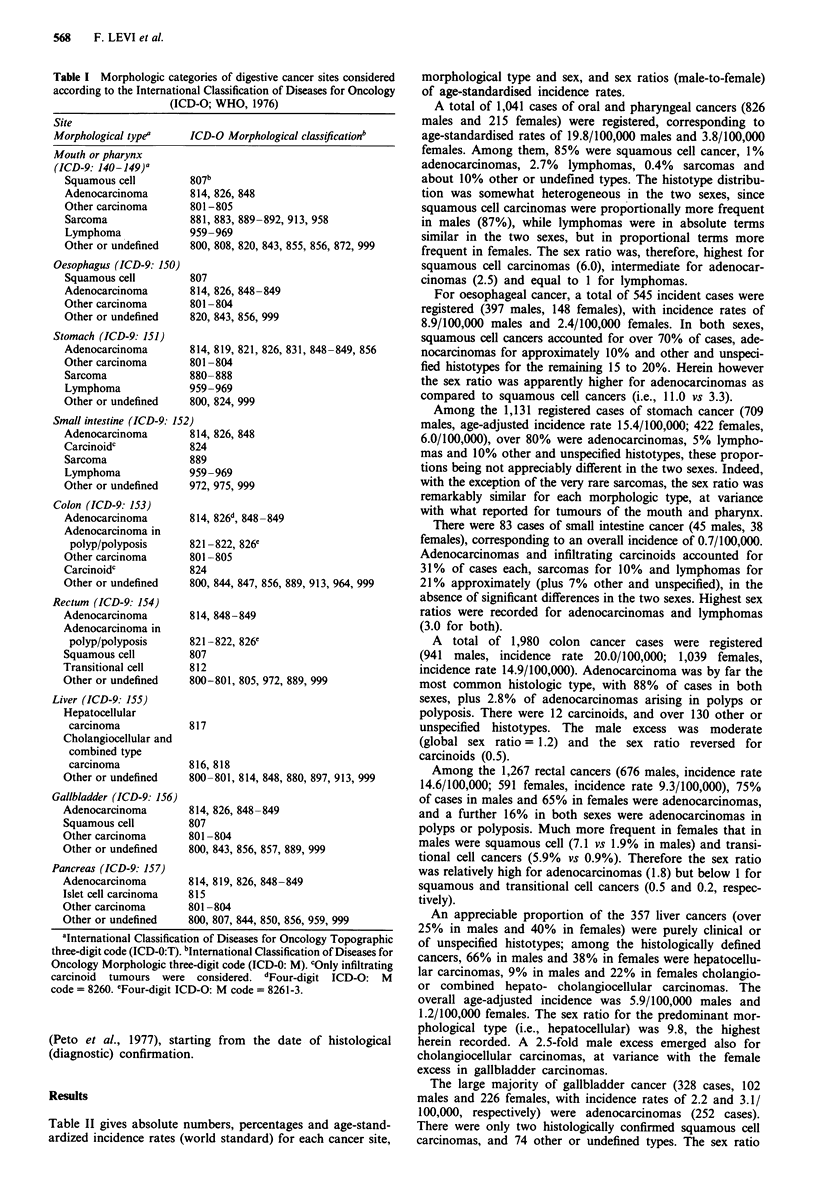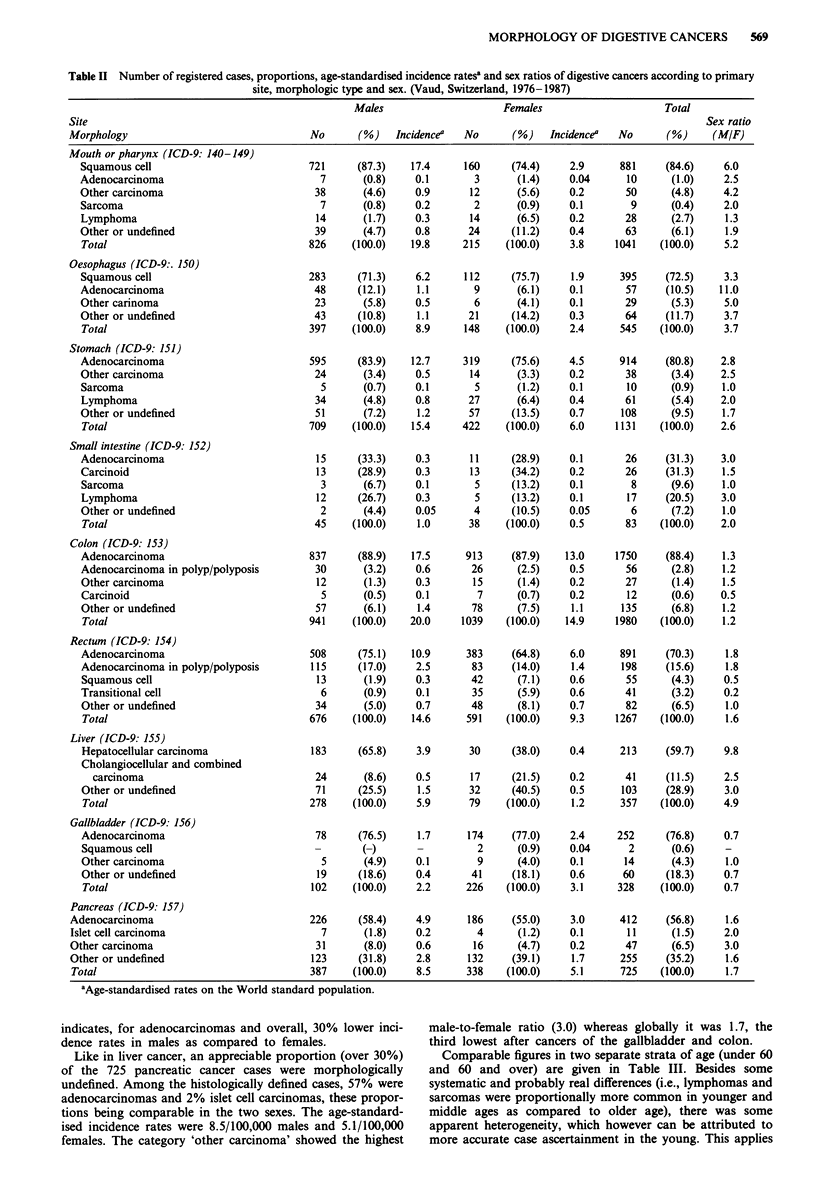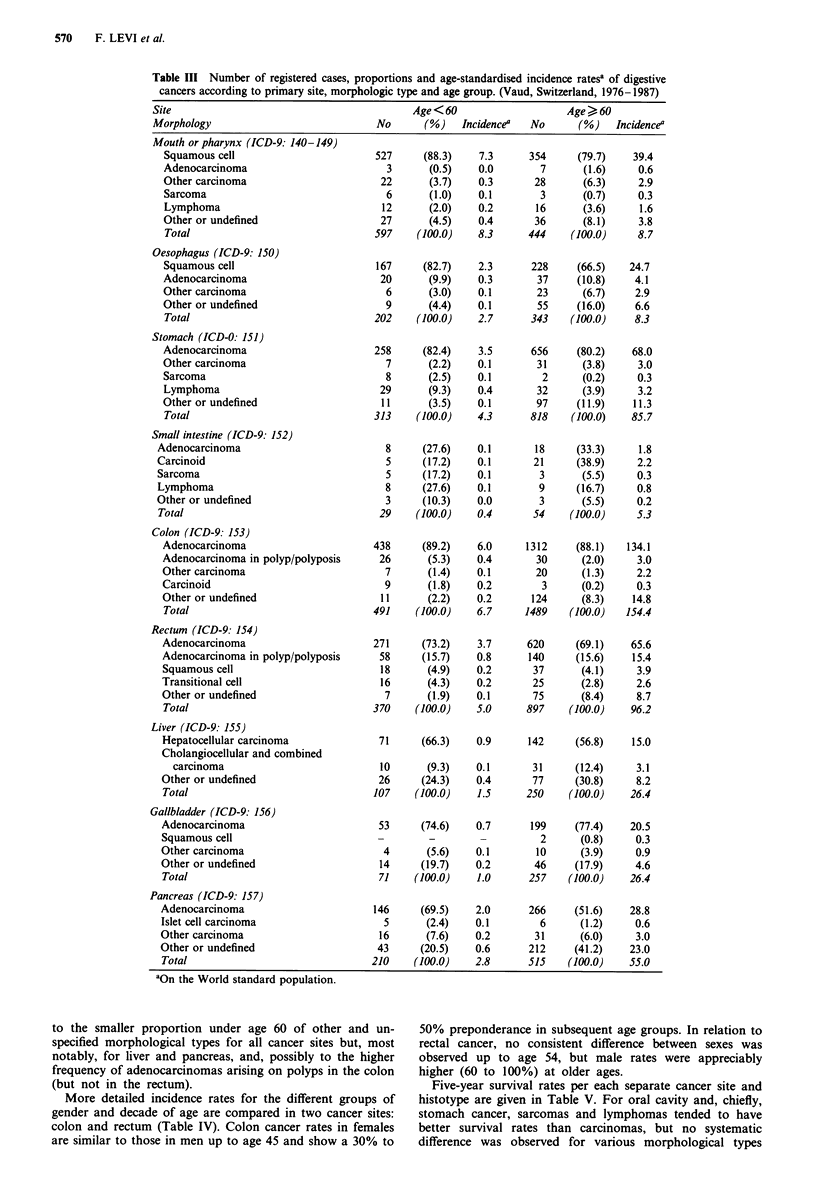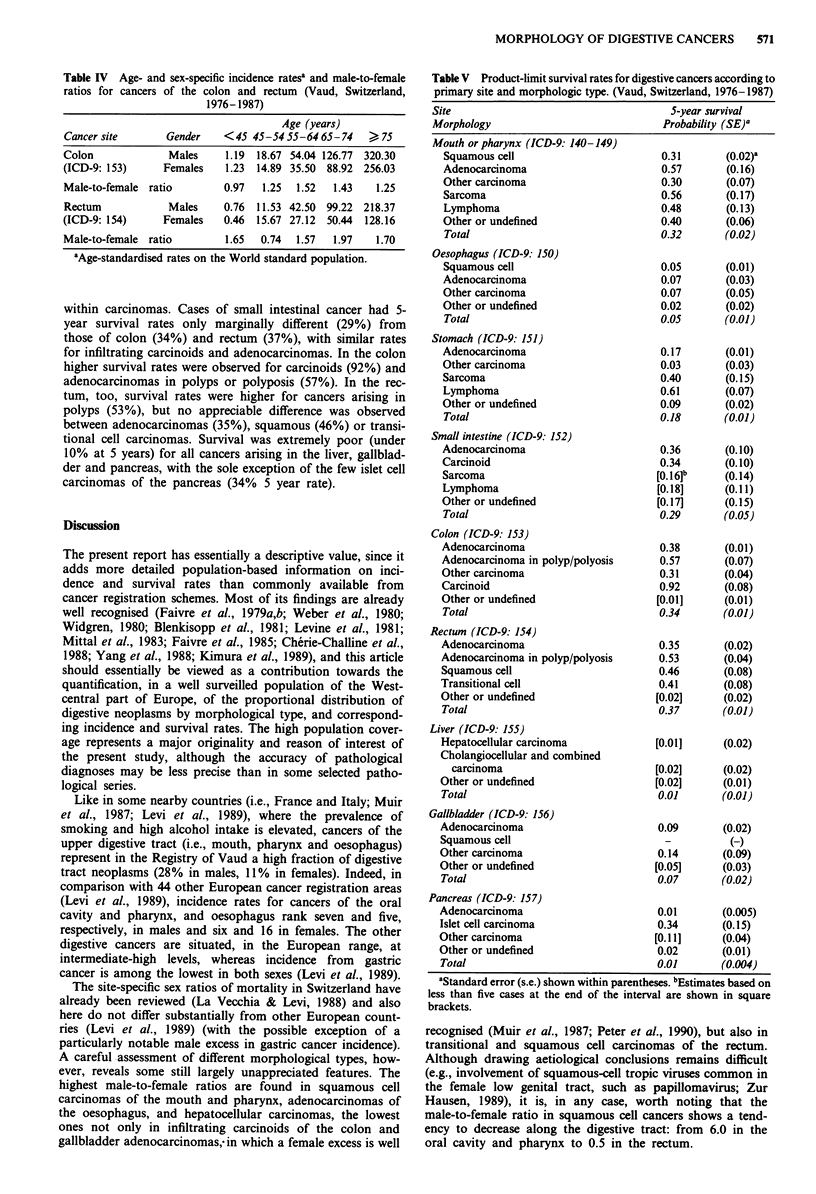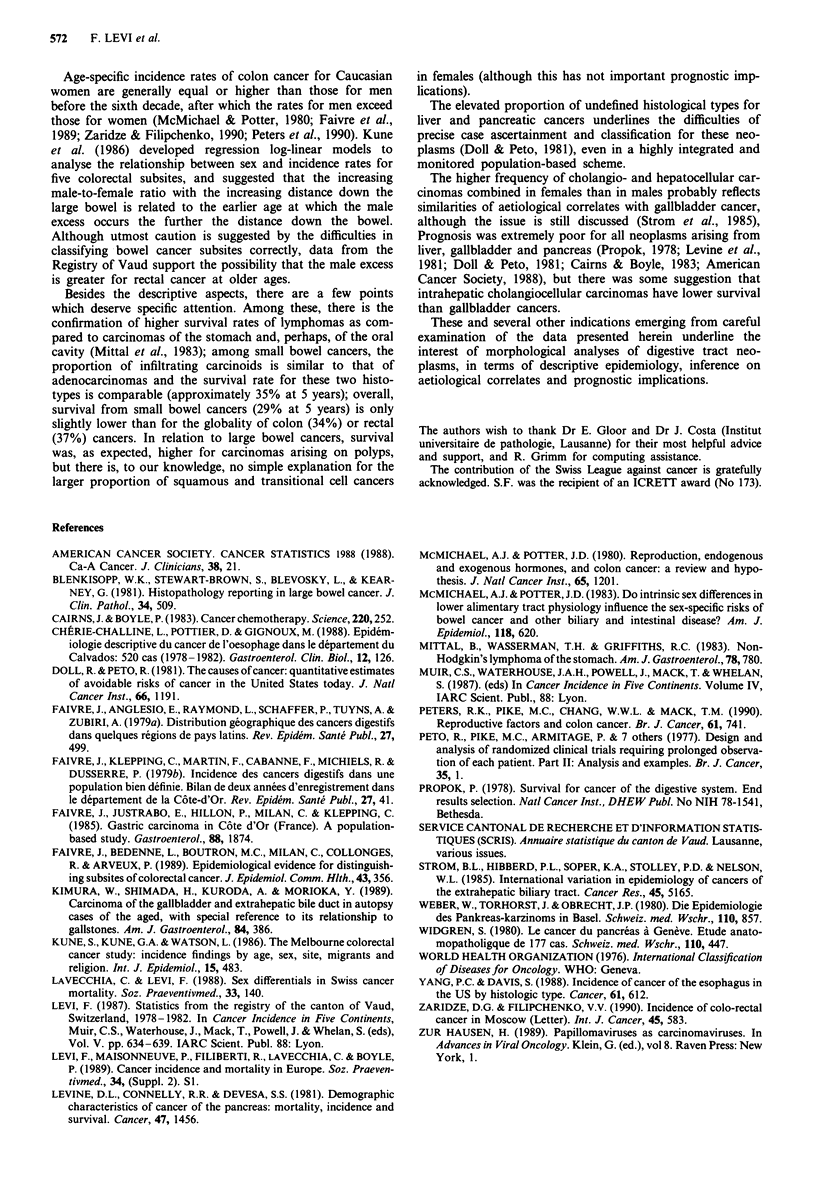# Morphologic analysis of digestive cancers from the registry of Vaud, Switzerland.

**DOI:** 10.1038/bjc.1991.132

**Published:** 1991-04

**Authors:** F. Levi, C. La Vecchia, S. Franceschi, V. C. Te

**Affiliations:** Registre vaudois des tumeurs, Institut universitaire de médecine sociale et préventive, Centre Hospitalier Universitaire Vaudois, Lausanne, Switzerland.

## Abstract

Detailed data and statistics per each morphological site of various digestive neoplasms were obtained for the period 1976-87 from the Vaud Cancer Registry datafile, a population-based cancer registration scheme covering about 530,000 inhabitants from the French-speaking part of Switzerland. Tabulations presented include absolute number of cases (1,041 oral and pharyngeal, 545 oesophageal, 1,131 gastric, 83 small intestine, 1,980 colon, 1,267 rectal, 357 liver, 328 gallbladder and 725 pancreatic cancers), percent distributions, age-standardised rates, sex ratios and 5-year survival. The report has essentially a descriptive value, and should be viewed as a contribution towards quantification, in a well surveilled population of the West-central part of Europe, of the proportional distribution of digestive neoplasms by morphological type, and corresponding incidence and survival rates. Among the points deserving specific attention, there are the elevated frequency of upper digestive tract cancers, the very high male-to-female ratios for squamous cell carcinomas, adenocarcinomas of the oesophagus and hepatocellular carcinomas of the liver, the female excesses in infiltrating carcinoids of the colon, transitional and squamous cell carcinomas of the rectum and adenocarcinomas of the gallbladder, and the crossover in male-to-female ratio in squamous cell carcinoma along the digestive tract (i.e. from 6.0 at the level of the mouth/pharynx to 0.5 in the rectum). As concerns survival, rates were higher for lymphomas and sarcomas than for carcinomas in oral cavity and stomach, similar for carcinoids and carcinomas in the small bowel (about 35% at 5 years), as well as for colon (34%) and rectal (37%) cancers. Some of the findings, such as the higher survival of carcinomas arising from polyps in the colon and rectum, or the higher proportion of cholangiocellular and combined cholangio- and hepatocellular carcinomas in females than in males find plausible prognostic or aetiologic correlates, but others, such as the large proportions of squamous and transitional cell cancers of the rectum in females are more difficult to explain. These and several other indications emerging from careful examination of the data herein presented underline the interest of morphological analyses of digestive tract cancers.


 © Macmillan Press Ltd., 1991

### Morphologic analysis of digestive cancers from the registry of Vaud, Switzerland

F. Levi[1,2], C. La Vecchia[2,3], S. Franceschi[4] & V.-C. Te[1]

[1]*Registre vaudois des tumeurs, Institut universitaire de médecine sociale et préventive, Centre Hospitalier Universitaire Vaudois, Falaises 1, 1011 Lausanne, Switzerland;* [2]*Institut universitaire de médecine sociale et préventive, Bugnon 17, 1005 Lausanne, Switzerland;* [3]*Istituto di Ricerche Farmacologiche 'Mario Negri', Via Eritrea 62, 20157 Milano, Italy; and* [4]*Servizio di Epidemiologia, Centro di Riferimento Oncologico, Via Pedemontana Occ, 33081 Aviano (PN), Italy.*

**Summary** Detailed data and statistics per each morphological site of various digestive neoplasms were obtained for the period 1976-87 from the Vaud Cancer Registry datafile, a population-based cancer registration scheme covering about 530,000 inhabitants from the French-speaking part of Switzerland. Tabulations presented include absolute number of cases (1,041 oral and pharyngeal, 545 oesophageal, 1,131 gastric, 83 small intestine, 1,980 colon, 1,267 rectal, 357 liver, 328 gallbladder and 725 pancreatic cancers), percent distributions, age-standardised rates, sex ratios and 5-year survival. The report has essentially a descriptive value, and should be viewed as a contribution towards quantification, in a well surveilled population of the West-central part of Europe, of the proportional distribution of digestive neoplasms by morphological type, and corresponding incidence and survival rates. Among the points deserving specific attention, there are the elevated frequency of upper digestive tract cancers, the very high male-to-female ratios for squamous cell carcinomas, adenocarcinomas of the oesophagus and hepatocellular carcinomas of the liver, the female excesses in infiltrating carcinoids of the colon, transitional and squamous cell carcinomas of the rectum and adenocarcinomas of the gallbladder, and the crossover in male-to-female ratio in squamous cell carcinoma along the digestive tract (i.e. from 6.0 at the level of the mouth/pharynx to 0.5 in the rectum). As concerns survival, rates were higher for lymphomas and sarcomas than for carcinomas in oral cavity and stomach, similar for carcinoids and carcinomas in the small bowel (about 35% at 5 years), as well as for colon (34%) and rectal (37%) cancers. Some of the findings, such as the higher survival of carcinomas arising from polyps in the colon and rectum, or the higher proportion of cholangiocellular and combined cholangio- and hepatocellular carcinomas in females than in males find plausible prognostic or aetiologic correlates, but others, such as the large proportions of squamous and transitional cell cancers of the rectum in females are more difficult to explain. These and several other indications emerging from careful examination of the data herein presented underline the interest of morphological analyses of digestive tract cancers.

In the scientific literature, cancer incidence registration and death certification statistics are usually reported by major sites (Muir *et al.*, 1987), while more detailed analyses by different histotypes or other morphological entities are confined to reports whose circulation is relatively limited.

Such information, however, has potential importance for epidemiological and clinical research (Faivre *et al.*, 1979*a*; Weber *et al.*, 1980; Widgren, 1980; Blenkisopp *et al.*, 1981; Levine *et al.*, 1981; Mittal *et al.*, 1983; Faivre *et al.*, 1985; Chérie-Challine *et al.*, 1988; Yang & Davis, 1989; Kimura *et al.*, 1989). For instance, various histotypes of the same cancer may recognise different aetiologies, and in a study or in a review of studies on risk factors, it is of interest to compare the distribution of histological types with that from population-based data. Similar problems may emerge in the analysis of clinical trials, since the morphological characteristics of a tumour may have important implications for evaluation of survival rates and, more in general, prognosis of the disease.

In the present report, therefore, we present absolute proportions, incidence, sex ratios and survival rates from the Cancer Registry of the Canton Vaud, Switzerland, for digestive sites, where detailed morphological distinctions and their variation according to age and sex has potential epidemiological and clinical relevance.

### Material and methods

The data included in the present analysis were derived from the Vaud Cancer Registry datafile, which includes data concerning incident cases of malignant neoplasms in the Canton (whose population in 1980 was about 530,000 inhabitants) (Levi, 1987). Population estimates are based on decennial censuses (1970, 1980), and on estimates by the Cantonal Office of Statistics for each calendar year and 5 year age group, based on official numbers of births, deaths, immigrations and emigrations (Service Cantonal de Recherche et d'Information Statistiques).

Notification is based on a voluntary agreement between the recording medical institutions of the Canton and the Registry. All hospitals, pathological laboratories and most practitioners are asked to report all new or past cases of cancer. The main source of notification is the Cantonal University Pathological Department of Lausanne which performs the majority of histological examinations for the population covered by the Registry. Most cases are registered repeatedly and from different institutions, thus ensuring completeness and accuracy of notification. Cases known only through the death certificate ('Death Certificate Only' cases (DCO)) contribute less than 5% of the average number of new cancer cases registered per year.

Information collected by the registry includes general demographic characteristics of the patient (age, sex, municipality of residence), site and histological type of the tumour according to the standard International Classification of Diseases for Oncology (ICD-O; Worth Health Organization, 1976), and time of registration. A total of 7,457 digestive invasive cancer cases, registered between 1976 and 1987, were included in the present study.

Digestive cancer sites and morphologic categories considered according to the ICD-O Classification are listed in Table I.

Passive and active follow-up information is recorded and each subsequent item of information concerning an already registered case is used to complete the record of that patient. Information coming from death certificates is routinely added to the morbidity file. The vital status of each registered case has thus been verified up to June 30, 1989.

Five-year crude survival probability and corresponding standard error were computed, for each topographical and morphological group, by means of the product-limit method

Correspondence: F. Levi, Registre vaudois des tumeurs, CHUV Falaises 1, 1011 Lausanne, Switzerland.
Received 29 August 1990; and in revised form 30 November 1990.

**Table I** Morphologic categories of digestive cancer sites considered according to the International Classification of Diseases for Oncology (ICD-O; WHO, 1976)

| *Site* *Morphological type*[a] | *ICD-O Morphological classification*[b] |
|---|---|
| *Mouth or pharynx (ICD-9: 140–149)*[a] | |
| Squamous cell | 807[b] |
| Adenocarcinoma | 814, 826, 848 |
| Other carcinoma | 801–805 |
| Sarcoma | 881, 883, 889–892, 913, 958 |
| Lymphoma | 959–969 |
| Other or undefined | 800, 808, 820, 843, 855, 856, 872, 999 |
| *Oesophagus (ICD-9: 150)* | |
| Squamous cell | 807 |
| Adenocarcinoma | 814, 826, 848–849 |
| Other carcinoma | 801–804 |
| Other or undefined | 820, 843, 856, 999 |
| *Stomach (ICD-9: 151)* | |
| Adenocarcinoma | 814, 819, 821, 826, 831, 848–849, 856 |
| Other carcinoma | 801–804 |
| Sarcoma | 880–888 |
| Lymphoma | 959–969 |
| Other or undefined | 800, 824, 999 |
| *Small intestine (ICD-9: 152)* | |
| Adenocarcinoma | 814, 826, 848 |
| Carcinoid[c] | 824 |
| Sarcoma | 889 |
| Lymphoma | 959–969 |
| Other or undefined | 972, 975, 999 |
| *Colon (ICD-9: 153)* | |
| Adenocarcinoma | 814, 826[d], 848–849 |
| Adenocarcinoma in polyp/polyposis | 821–822, 826[e] |
| Other carcinoma | 801–805 |
| Carcinoid[c] | 824 |
| Other or undefined | 800, 844, 847, 856, 889, 913, 964, 999 |
| *Rectum (ICD-9: 154)* | |
| Adenocarcinoma | 814, 848–849 |
| Adenocarcinoma in polyp/polyposis | 821–822, 826[e] |
| Squamous cell | 807 |
| Transitional cell | 812 |
| Other or undefined | 800–801, 805, 972, 889, 999 |
| *Liver (ICD-9: 155)* | |
| Hepatocellular carcinoma | 817 |
| Cholangiocellular and combined type carcinoma | 816, 818 |
| Other or undefined | 800–801, 814, 848, 880, 897, 913, 999 |
| *Gallbladder (ICD-9: 156)* | |
| Adenocarcinoma | 814, 826, 848–849 |
| Squamous cell | 807 |
| Other carcinoma | 801–804 |
| Other or undefined | 800, 843, 856, 857, 889, 999 |
| *Pancreas (ICD-9: 157)* | |
| Adenocarcinoma | 814, 819, 826, 848–849 |
| Islet cell carcinoma | 815 |
| Other carcinoma | 801–804 |
| Other or undefined | 800, 807, 844, 850, 856, 959, 999 |

[a]International Classification of Diseases for Oncology Topographic three-digit code (ICD-0:T). [b]International Classification of Diseases for Oncology Morphologic three-digit code (ICD-0: M). [c]Only infiltrating carcinoid tumours were considered. [d]Four-digit ICD-O: M code = 8260. [e]Four-digit ICD-O: M code = 8261-3.

(Peto *et al.*, 1977), starting from the date of histological (diagnostic) confirmation.

### Results

Table II gives absolute numbers, percentages and age-standardized incidence rates (world standard) for each cancer site, morphological type and sex, and sex ratios (male-to-female) of age-standardised incidence rates.

A total of 1,041 cases of oral and pharyngeal cancers (826 males and 215 females) were registered, corresponding to age-standardised rates of 19.8/100,000 males and 3.8/100,000 females. Among them, 85% were squamous cell cancer, 1% adenocarcinomas, 2.7% lymphomas, 0.4% sarcomas and about 10% other or undefined types. The histotype distribution was somewhat heterogeneous in the two sexes, since squamous cell carcinomas were proportionally more frequent in males (87%), while lymphomas were in absolute terms similar in the two sexes, but in proportional terms more frequent in females. The sex ratio was, therefore, highest for squamous cell carcinomas (6.0), intermediate for adenocarcinomas (2.5) and equal to 1 for lymphomas.

For oesophageal cancer, a total of 545 incident cases were registered (397 males, 148 females), with incidence rates of 8.9/100,000 males and 2.4/100,000 females. In both sexes, squamous cell cancers accounted for over 70% of cases, adenocarcinomas for approximately 10% and other and unspecified histotypes for the remaining 15 to 20%. Herein however the sex ratio was apparently higher for adenocarcinomas as compared to squamous cell cancers (i.e., 11.0 *vs* 3.3).

Among the 1,131 registered cases of stomach cancer (709 males, age-adjusted incidence rate 15.4/100,000; 422 females, 6.0/100,000), over 80% were adenocarcinomas, 5% lymphomas and 10% other and unspecified histotypes, these proportions being not appreciably different in the two sexes. Indeed, with the exception of the very rare sarcomas, the sex ratio was remarkably similar for each morphologic type, at variance with what reported for tumours of the mouth and pharynx.

There were 83 cases of small intestine cancer (45 males, 38 females), corresponding to an overall incidence of 0.7/100,000. Adenocarcinomas and infiltrating carcinoids accounted for 31% of cases each, sarcomas for 10% and lymphomas for 21% approximately (plus 7% other and unspecified), in the absence of significant differences in the two sexes. Highest sex ratios were recorded for adenocarcinomas and lymphomas (3.0 for both).

A total of 1,980 colon cancer cases were registered (941 males, incidence rate 20.0/100,000; 1,039 females, incidence rate 14.9/100,000). Adenocarcinoma was by far the most common histologic type, with 88% of cases in both sexes, plus 2.8% of adenocarcinomas arising in polyps or polyposis. There were 12 carcinoids, and over 130 other or unspecified histotypes. The male excess was moderate (global sex ratio = 1.2) and the sex ratio reversed for carcinoids (0.5).

Among the 1,267 rectal cancers (676 males, incidence rate 14.6/100,000; 591 females, incidence rate 9.3/100,000), 75% of cases in males and 65% in females were adenocarcinomas, and a further 16% in both sexes were adenocarcinomas in polyps or polyposis. Much more frequent in females that in males were squamous cell (7.1 *vs* 1.9% in males) and transitional cell cancers (5.9% *vs* 0.9%). Therefore the sex ratio was relatively high for adenocarcinomas (1.8) but below 1 for squamous and transitional cell cancers (0.5 and 0.2, respectively).

An appreciable proportion of the 357 liver cancers (over 25% in males and 40% in females) were purely clinical or of unspecified histotypes; among the histologically defined cancers, 66% in males and 38% in females were hepatocellular carcinomas, 9% in males and 22% in females cholangio- or combined hepato- cholangiocellular carcinomas. The overall age-adjusted incidence was 5.9/100,000 males and 1.2/100,000 females. The sex ratio for the predominant morphological type (i.e., hepatocellular) was 9.8, the highest herein recorded. A 2.5-fold male excess emerged also for cholangiocellular carcinomas, at variance with the female excess in gallbladder carcinomas.

The large majority of gallbladder cancer (328 cases, 102 males and 226 females, with incidence rates of 2.2 and 3.1/100,000, respectively) were adenocarcinomas (252 cases). There were only two histologically confirmed squamous cell carcinomas, and 74 other or undefined types. The sex ratio

**Table II** Number of registered cases, proportions, age-standardised incidence rates[a] and sex ratios of digestive cancers according to primary site, morphologic type and sex. (Vaud, Switzerland, 1976–1987)

| Site / Morphology | Males No | Males (%) | Males Incidence[a] | Females No | Females (%) | Females Incidence[a] | Total No | Total (%) | Sex ratio (M/F) |
|---|---|---|---|---|---|---|---|---|---|
| *Mouth or pharynx (ICD-9: 140–149)* | | | | | | | | | |
| Squamous cell | 721 | (87.3) | 17.4 | 160 | (74.4) | 2.9 | 881 | (84.6) | 6.0 |
| Adenocarcinoma | 7 | (0.8) | 0.1 | 3 | (1.4) | 0.04 | 10 | (1.0) | 2.5 |
| Other carcinoma | 38 | (4.6) | 0.9 | 12 | (5.6) | 0.2 | 50 | (4.8) | 4.2 |
| Sarcoma | 7 | (0.8) | 0.2 | 2 | (0.9) | 0.1 | 9 | (0.4) | 2.0 |
| Lymphoma | 14 | (1.7) | 0.3 | 14 | (6.5) | 0.2 | 28 | (2.7) | 1.3 |
| Other or undefined | 39 | (4.7) | 0.8 | 24 | (11.2) | 0.4 | 63 | (6.1) | 1.9 |
| *Total* | 826 | (100.0) | 19.8 | 215 | (100.0) | 3.8 | 1041 | (100.0) | 5.2 |
| *Oesophagus (ICD-9:. 150)* | | | | | | | | | |
| Squamous cell | 283 | (71.3) | 6.2 | 112 | (75.7) | 1.9 | 395 | (72.5) | 3.3 |
| Adenocarcinoma | 48 | (12.1) | 1.1 | 9 | (6.1) | 0.1 | 57 | (10.5) | 11.0 |
| Other carinoma | 23 | (5.8) | 0.5 | 6 | (4.1) | 0.1 | 29 | (5.3) | 5.0 |
| Other or undefined | 43 | (10.8) | 1.1 | 21 | (14.2) | 0.3 | 64 | (11.7) | 3.7 |
| *Total* | 397 | (100.0) | 8.9 | 148 | (100.0) | 2.4 | 545 | (100.0) | 3.7 |
| *Stomach (ICD-9: 151)* | | | | | | | | | |
| Adenocarcinoma | 595 | (83.9) | 12.7 | 319 | (75.6) | 4.5 | 914 | (80.8) | 2.8 |
| Other carcinoma | 24 | (3.4) | 0.5 | 14 | (3.3) | 0.2 | 38 | (3.4) | 2.5 |
| Sarcoma | 5 | (0.7) | 0.1 | 5 | (1.2) | 0.1 | 10 | (0.9) | 1.0 |
| Lymphoma | 34 | (4.8) | 0.8 | 27 | (6.4) | 0.4 | 61 | (5.4) | 2.0 |
| Other or undefined | 51 | (7.2) | 1.2 | 57 | (13.5) | 0.7 | 108 | (9.5) | 1.7 |
| *Total* | 709 | (100.0) | 15.4 | 422 | (100.0) | 6.0 | 1131 | (100.0) | 2.6 |
| *Small intestine (ICD-9: 152)* | | | | | | | | | |
| Adenocarcinoma | 15 | (33.3) | 0.3 | 11 | (28.9) | 0.1 | 26 | (31.3) | 3.0 |
| Carcinoid | 13 | (28.9) | 0.3 | 13 | (34.2) | 0.2 | 26 | (31.3) | 1.5 |
| Sarcoma | 3 | (6.7) | 0.1 | 5 | (13.2) | 0.1 | 8 | (9.6) | 1.0 |
| Lymphoma | 12 | (26.7) | 0.3 | 5 | (13.2) | 0.1 | 17 | (20.5) | 3.0 |
| Other or undefined | 2 | (4.4) | 0.05 | 4 | (10.5) | 0.05 | 6 | (7.2) | 1.0 |
| *Total* | 45 | (100.0) | 1.0 | 38 | (100.0) | 0.5 | 83 | (100.0) | 2.0 |
| *Colon (ICD-9: 153)* | | | | | | | | | |
| Adenocarcinoma | 837 | (88.9) | 17.5 | 913 | (87.9) | 13.0 | 1750 | (88.4) | 1.3 |
| Adenocarcinoma in polyp/polyposis | 30 | (3.2) | 0.6 | 26 | (2.5) | 0.5 | 56 | (2.8) | 1.2 |
| Other carcinoma | 12 | (1.3) | 0.3 | 15 | (1.4) | 0.2 | 27 | (1.4) | 1.5 |
| Carcinoid | 5 | (0.5) | 0.1 | 7 | (0.7) | 0.2 | 12 | (0.6) | 0.5 |
| Other or undefined | 57 | (6.1) | 1.4 | 78 | (7.5) | 1.1 | 135 | (6.8) | 1.2 |
| *Total* | 941 | (100.0) | 20.0 | 1039 | (100.0) | 14.9 | 1980 | (100.0) | 1.2 |
| *Rectum (ICD-9: 154)* | | | | | | | | | |
| Adenocarcinoma | 508 | (75.1) | 10.9 | 383 | (64.8) | 6.0 | 891 | (70.3) | 1.8 |
| Adenocarcinoma in polyp/polyposis | 115 | (17.0) | 2.5 | 83 | (14.0) | 1.4 | 198 | (15.6) | 1.8 |
| Squamous cell | 13 | (1.9) | 0.3 | 42 | (7.1) | 0.6 | 55 | (4.3) | 0.5 |
| Transitional cell | 6 | (0.9) | 0.1 | 35 | (5.9) | 0.6 | 41 | (3.2) | 0.2 |
| Other or undefined | 34 | (5.0) | 0.7 | 48 | (8.1) | 0.7 | 82 | (6.5) | 1.0 |
| *Total* | 676 | (100.0) | 14.6 | 591 | (100.0) | 9.3 | 1267 | (100.0) | 1.6 |
| *Liver (ICD-9: 155)* | | | | | | | | | |
| Hepatocellular carcinoma | 183 | (65.8) | 3.9 | 30 | (38.0) | 0.4 | 213 | (59.7) | 9.8 |
| Cholangiocellular and combined carcinoma | 24 | (8.6) | 0.5 | 17 | (21.5) | 0.2 | 41 | (11.5) | 2.5 |
| Other or undefined | 71 | (25.5) | 1.5 | 32 | (40.5) | 0.5 | 103 | (28.9) | 3.0 |
| *Total* | 278 | (100.0) | 5.9 | 79 | (100.0) | 1.2 | 357 | (100.0) | 4.9 |
| *Gallbladder (ICD-9: 156)* | | | | | | | | | |
| Adenocarcinoma | 78 | (76.5) | 1.7 | 174 | (77.0) | 2.4 | 252 | (76.8) | 0.7 |
| Squamous cell | – | (–) | – | 2 | (0.9) | 0.04 | 2 | (0.6) | – |
| Other carcinoma | 5 | (4.9) | 0.1 | 9 | (4.0) | 0.1 | 14 | (4.3) | 1.0 |
| Other or undefined | 19 | (18.6) | 0.4 | 41 | (18.1) | 0.6 | 60 | (18.3) | 0.7 |
| *Total* | 102 | (100.0) | 2.2 | 226 | (100.0) | 3.1 | 328 | (100.0) | 0.7 |
| *Pancreas (ICD-9: 157)* | | | | | | | | | |
| Adenocarcinoma | 226 | (58.4) | 4.9 | 186 | (55.0) | 3.0 | 412 | (56.8) | 1.6 |
| Islet cell carcinoma | 7 | (1.8) | 0.2 | 4 | (1.2) | 0.1 | 11 | (1.5) | 2.0 |
| Other carcinoma | 31 | (8.0) | 0.6 | 16 | (4.7) | 0.2 | 47 | (6.5) | 3.0 |
| Other or undefined | 123 | (31.8) | 2.8 | 132 | (39.1) | 1.7 | 255 | (35.2) | 1.6 |
| *Total* | 387 | (100.0) | 8.5 | 338 | (100.0) | 5.1 | 725 | (100.0) | 1.7 |

[a]Age-standardised rates on the World standard population.

indicates, for adenocarcinomas and overall, 30% lower incidence rates in males as compared to females.

Like in liver cancer, an appreciable proportion (over 30%) of the 725 pancreatic cancer cases were morphologically undefined. Among the histologically defined cases, 57% were adenocarcinomas and 2% islet cell carcinomas, these proportions being comparable in the two sexes. The age-standardised incidence rates were 8.5/100,000 males and 5.1/100,000 females. The category 'other carcinoma' showed the highest male-to-female ratio (3.0) whereas globally it was 1.7, the third lowest after cancers of the gallbladder and colon.

Comparable figures in two separate strata of age (under 60 and 60 and over) are given in Table III. Besides some systematic and probably real differences (i.e., lymphomas and sarcomas were proportionally more common in younger and middle ages as compared to older age), there was some apparent heterogeneity, which however can be attributed to more accurate case ascertainment in the young. This applies

**Table III** Number of registered cases, proportions and age-standardised incidence rates[a] of digestive cancers according to primary site, morphologic type and age group. (Vaud, Switzerland, 1976–1987)

| *Site* / *Morphology* | Age<60 *No* | Age<60 *(%)* | Age<60 *Incidence[a]* | Age≥60 *No* | Age≥60 *(%)* | Age≥60 *Incidence[a]* |
|---|---|---|---|---|---|---|
| *Mouth or pharynx (ICD-9: 140–149)* | | | | | | |
| Squamous cell | 527 | (88.3) | 7.3 | 354 | (79.7) | 39.4 |
| Adenocarcinoma | 3 | (0.5) | 0.0 | 7 | (1.6) | 0.6 |
| Other carcinoma | 22 | (3.7) | 0.3 | 28 | (6.3) | 2.9 |
| Sarcoma | 6 | (1.0) | 0.1 | 3 | (0.7) | 0.3 |
| Lymphoma | 12 | (2.0) | 0.2 | 16 | (3.6) | 1.6 |
| Other or undefined | 27 | (4.5) | 0.4 | 36 | (8.1) | 3.8 |
| *Total* | *597* | *(100.0)* | *8.3* | *444* | *(100.0)* | *8.7* |
| *Oesophagus (ICD-9: 150)* | | | | | | |
| Squamous cell | 167 | (82.7) | 2.3 | 228 | (66.5) | 24.7 |
| Adenocarcinoma | 20 | (9.9) | 0.3 | 37 | (10.8) | 4.1 |
| Other carcinoma | 6 | (3.0) | 0.1 | 23 | (6.7) | 2.9 |
| Other or undefined | 9 | (4.4) | 0.1 | 55 | (16.0) | 6.6 |
| *Total* | *202* | *(100.0)* | *2.7* | *343* | *(100.0)* | *8.3* |
| *Stomach (ICD-0: 151)* | | | | | | |
| Adenocarcinoma | 258 | (82.4) | 3.5 | 656 | (80.2) | 68.0 |
| Other carcinoma | 7 | (2.2) | 0.1 | 31 | (3.8) | 3.0 |
| Sarcoma | 8 | (2.5) | 0.1 | 2 | (0.2) | 0.3 |
| Lymphoma | 29 | (9.3) | 0.4 | 32 | (3.9) | 3.2 |
| Other or undefined | 11 | (3.5) | 0.1 | 97 | (11.9) | 11.3 |
| *Total* | *313* | *(100.0)* | *4.3* | *818* | *(100.0)* | *85.7* |
| *Small intestine (ICD-9: 152)* | | | | | | |
| Adenocarcinoma | 8 | (27.6) | 0.1 | 18 | (33.3) | 1.8 |
| Carcinoid | 5 | (17.2) | 0.1 | 21 | (38.9) | 2.2 |
| Sarcoma | 5 | (17.2) | 0.1 | 3 | (5.5) | 0.3 |
| Lymphoma | 8 | (27.6) | 0.1 | 9 | (16.7) | 0.8 |
| Other or undefined | 3 | (10.3) | 0.0 | 3 | (5.5) | 0.2 |
| *Total* | *29* | *(100.0)* | *0.4* | *54* | *(100.0)* | *5.3* |
| *Colon (ICD-9: 153)* | | | | | | |
| Adenocarcinoma | 438 | (89.2) | 6.0 | 1312 | (88.1) | 134.1 |
| Adenocarcinoma in polyp/polyposis | 26 | (5.3) | 0.4 | 30 | (2.0) | 3.0 |
| Other carcinoma | 7 | (1.4) | 0.1 | 20 | (1.3) | 2.2 |
| Carcinoid | 9 | (1.8) | 0.2 | 3 | (0.2) | 0.3 |
| Other or undefined | 11 | (2.2) | 0.2 | 124 | (8.3) | 14.8 |
| *Total* | *491* | *(100.0)* | *6.7* | *1489* | *(100.0)* | *154.4* |
| *Rectum (ICD-9: 154)* | | | | | | |
| Adenocarcinoma | 271 | (73.2) | 3.7 | 620 | (69.1) | 65.6 |
| Adenocarcinoma in polyp/polyposis | 58 | (15.7) | 0.8 | 140 | (15.6) | 15.4 |
| Squamous cell | 18 | (4.9) | 0.2 | 37 | (4.1) | 3.9 |
| Transitional cell | 16 | (4.3) | 0.2 | 25 | (2.8) | 2.6 |
| Other or undefined | 7 | (1.9) | 0.1 | 75 | (8.4) | 8.7 |
| *Total* | *370* | *(100.0)* | *5.0* | *897* | *(100.0)* | *96.2* |
| *Liver (ICD-9: 155)* | | | | | | |
| Hepatocellular carcinoma | 71 | (66.3) | 0.9 | 142 | (56.8) | 15.0 |
| Cholangiocellular and combined carcinoma | 10 | (9.3) | 0.1 | 31 | (12.4) | 3.1 |
| Other or undefined | 26 | (24.3) | 0.4 | 77 | (30.8) | 8.2 |
| *Total* | *107* | *(100.0)* | *1.5* | *250* | *(100.0)* | *26.4* |
| *Gallbladder (ICD-9: 156)* | | | | | | |
| Adenocarcinoma | 53 | (74.6) | 0.7 | 199 | (77.4) | 20.5 |
| Squamous cell | – | – | – | 2 | (0.8) | 0.3 |
| Other carcinoma | 4 | (5.6) | 0.1 | 10 | (3.9) | 0.9 |
| Other or undefined | 14 | (19.7) | 0.2 | 46 | (17.9) | 4.6 |
| *Total* | *71* | *(100.0)* | *1.0* | *257* | *(100.0)* | *26.4* |
| *Pancreas (ICD-9: 157)* | | | | | | |
| Adenocarcinoma | 146 | (69.5) | 2.0 | 266 | (51.6) | 28.8 |
| Islet cell carcinoma | 5 | (2.4) | 0.1 | 6 | (1.2) | 0.6 |
| Other carcinoma | 16 | (7.6) | 0.2 | 31 | (6.0) | 3.0 |
| Other or undefined | 43 | (20.5) | 0.6 | 212 | (41.2) | 23.0 |
| *Total* | *210* | *(100.0)* | *2.8* | *515* | *(100.0)* | *55.0* |

[a]On the World standard population.

to the smaller proportion under age 60 of other and unspecified morphological types for all cancer sites but, most notably, for liver and pancreas, and, possibly to the higher frequency of adenocarcinomas arising on polyps in the colon (but not in the rectum).

More detailed incidence rates for the different groups of gender and decade of age are compared in two cancer sites: colon and rectum (Table IV). Colon cancer rates in females are similar to those in men up to age 45 and show a 30% to 50% preponderance in subsequent age groups. In relation to rectal cancer, no consistent difference between sexes was observed up to age 54, but male rates were appreciably higher (60 to 100%) at older ages.

Five-year survival rates per each separate cancer site and histotype are given in Table V. For oral cavity and, chiefly, stomach cancer, sarcomas and lymphomas tended to have better survival rates than carcinomas, but no systematic difference was observed for various morphological types

**Table IV** Age- and sex-specific incidence rates[a] and male-to-female ratios for cancers of the colon and rectum (Vaud, Switzerland, 1976–1987)

| Cancer site | Gender | Age (years) <45 | 45–54 | 55–64 | 65–74 | ≥75 |
|---|---|---|---|---|---|---|
| Colon | Males | 1.19 | 18.67 | 54.04 | 126.77 | 320.30 |
| (ICD-9: 153) | Females | 1.23 | 14.89 | 35.50 | 88.92 | 256.03 |
| Male-to-female ratio | | 0.97 | 1.25 | 1.52 | 1.43 | 1.25 |
| Rectum | Males | 0.76 | 11.53 | 42.50 | 99.22 | 218.37 |
| (ICD-9: 154) | Females | 0.46 | 15.67 | 27.12 | 50.44 | 128.16 |
| Male-to-female ratio | | 1.65 | 0.74 | 1.57 | 1.97 | 1.70 |

[a]Age-standardised rates on the World standard population.

within carcinomas. Cases of small intestinal cancer had 5-year survival rates only marginally different (29%) from those of colon (34%) and rectum (37%), with similar rates for infiltrating carcinoids and adenocarcinomas. In the colon higher survival rates were observed for carcinoids (92%) and adenocarcinomas in polyps or polyposis (57%). In the rectum, too, survival rates were higher for cancers arising in polyps (53%), but no appreciable difference was observed between adenocarcinomas (35%), squamous (46%) or transitional cell carcinomas. Survival was extremely poor (under 10% at 5 years) for all cancers arising in the liver, gallbladder and pancreas, with the sole exception of the few islet cell carcinomas of the pancreas (34% 5 year rate).

### Discussion

The present report has essentially a descriptive value, since it adds more detailed population-based information on incidence and survival rates than commonly available from cancer registration schemes. Most of its findings are already well recognised (Faivre *et al.*, 1979*a*,*b*; Weber *et al.*, 1980; Widgren, 1980; Blenkisopp *et al.*, 1981; Levine *et al.*, 1981; Mittal *et al.*, 1983; Faivre *et al.*, 1985; Chérie-Challine *et al.*, 1988; Yang *et al.*, 1988; Kimura *et al.*, 1989), and this article should essentially be viewed as a contribution towards the quantification, in a well surveilled population of the West-central part of Europe, of the proportional distribution of digestive neoplasms by morphological type, and corresponding incidence and survival rates. The high population coverage represents a major originality and reason of interest of the present study, although the accuracy of pathological diagnoses may be less precise than in some selected pathological series.

Like in some nearby countries (i.e., France and Italy; Muir *et al.*, 1987; Levi *et al.*, 1989), where the prevalence of smoking and high alcohol intake is elevated, cancers of the upper digestive tract (i.e., mouth, pharynx and oesophagus) represent in the Registry of Vaud a high fraction of digestive tract neoplasms (28% in males, 11% in females). Indeed, in comparison with 44 other European cancer registration areas (Levi *et al.*, 1989), incidence rates for cancers of the oral cavity and pharynx, and oesophagus rank seven and five, respectively, in males and six and 16 in females. The other digestive cancers are situated, in the European range, at intermediate-high levels, whereas incidence from gastric cancer is among the lowest in both sexes (Levi *et al.*, 1989).

The site-specific sex ratios of mortality in Switzerland have already been reviewed (La Vecchia & Levi, 1988) and also here do not differ substantially from other European countries (Levi *et al.*, 1989) (with the possible exception of a particularly notable male excess in gastric cancer incidence). A careful assessment of different morphological types, however, reveals some still largely unappreciated features. The highest male-to-female ratios are found in squamous cell carcinomas of the mouth and pharynx, adenocarcinomas of the oesophagus, and hepatocellular carcinomas, the lowest ones not only in infiltrating carcinoids of the colon and gallbladder adenocarcinomas, in which a female excess is well recognised (Muir *et al.*, 1987; Peter *et al.*, 1990), but also in transitional and squamous cell carcinomas of the rectum. Although drawing aetiological conclusions remains difficult (e.g., involvement of squamous-cell tropic viruses common in the female low genital tract, such as papillomavirus; Zur Hausen, 1989), it is, in any case, worth noting that the male-to-female ratio in squamous cell cancers shows a tendency to decrease along the digestive tract: from 6.0 in the oral cavity and pharynx to 0.5 in the rectum.

**Table V** Product-limit survival rates for digestive cancers according to primary site and morphologic type. (Vaud, Switzerland, 1976–1987)

| *Site* / *Morphology* | *5-year survival Probability* | *(SE)[a]* |
|---|---|---|
| *Mouth or pharynx (ICD-9: 140–149)* | | |
| Squamous cell | 0.31 | (0.02)[a] |
| Adenocarcinoma | 0.57 | (0.16) |
| Other carcinoma | 0.30 | (0.07) |
| Sarcoma | 0.56 | (0.17) |
| Lymphoma | 0.48 | (0.13) |
| Other or undefined | 0.40 | (0.06) |
| *Total* | *0.32* | *(0.02)* |
| *Oesophagus (ICD-9: 150)* | | |
| Squamous cell | 0.05 | (0.01) |
| Adenocarcinoma | 0.07 | (0.03) |
| Other carcinoma | 0.07 | (0.05) |
| Other or undefined | 0.02 | (0.02) |
| *Total* | *0.05* | *(0.01)* |
| *Stomach (ICD-9: 151)* | | |
| Adenocarcinoma | 0.17 | (0.01) |
| Other carcinoma | 0.03 | (0.03) |
| Sarcoma | 0.40 | (0.15) |
| Lymphoma | 0.61 | (0.07) |
| Other or undefined | 0.09 | (0.02) |
| *Total* | *0.18* | *(0.01)* |
| *Small intestine (ICD-9: 152)* | | |
| Adenocarcinoma | 0.36 | (0.10) |
| Carcinoid | 0.34 | (0.10) |
| Sarcoma | [0.16][b] | (0.14) |
| Lymphoma | [0.18] | (0.11) |
| Other or undefined | [0.17] | (0.15) |
| *Total* | *0.29* | *(0.05)* |
| *Colon (ICD-9: 153)* | | |
| Adenocarcinoma | 0.38 | (0.01) |
| Adenocarcinoma in polyp/polyosis | 0.57 | (0.07) |
| Other carcinoma | 0.31 | (0.04) |
| Carcinoid | 0.92 | (0.08) |
| Other or undefined | [0.01] | (0.01) |
| *Total* | *0.34* | *(0.01)* |
| *Rectum (ICD-9: 154)* | | |
| Adenocarcinoma | 0.35 | (0.02) |
| Adenocarcinoma in polyp/polyosis | 0.53 | (0.04) |
| Squamous cell | 0.46 | (0.08) |
| Transitional cell | 0.41 | (0.08) |
| Other or undefined | [0.02] | (0.02) |
| *Total* | *0.37* | *(0.01)* |
| *Liver (ICD-9: 155)* | | |
| Hepatocellular carcinoma | [0.01] | (0.02) |
| Cholangiocellular and combined carcinoma | [0.02] | (0.02) |
| Other or undefined | [0.02] | (0.01) |
| *Total* | *0.01* | *(0.01)* |
| *Gallbladder (ICD-9: 156)* | | |
| Adenocarcinoma | 0.09 | (0.02) |
| Squamous cell | – | (–) |
| Other carcinoma | 0.14 | (0.09) |
| Other or undefined | [0.05] | (0.03) |
| *Total* | *0.07* | *(0.02)* |
| *Pancreas (ICD-9: 157)* | | |
| Adenocarcinoma | 0.01 | (0.005) |
| Islet cell carcinoma | 0.34 | (0.15) |
| Other carcinoma | [0.11] | (0.04) |
| Other or undefined | 0.02 | (0.01) |
| *Total* | *0.01* | *(0.004)* |

[a]Standard error (s.e.) shown within parentheses. [b]Estimates based on less than five cases at the end of the interval are shown in square brackets.

Age-specific incidence rates of colon cancer for Caucasian women are generally equal or higher than those for men before the sixth decade, after which the rates for men exceed those for women (McMichael & Potter, 1980; Faivre et al., 1989; Zaridze & Filipchenko, 1990; Peters et al., 1990). Kune et al. (1986) developed regression log-linear models to analyse the relationship between sex and incidence rates for five colorectal subsites, and suggested that the increasing male-to-female ratio with the increasing distance down the large bowel is related to the earlier age at which the male excess occurs the further the distance down the bowel. Although utmost caution is suggested by the difficulties in classifying bowel cancer subsites correctly, data from the Registry of Vaud support the possibility that the male excess is greater for rectal cancer at older ages.

Besides the descriptive aspects, there are a few points which deserve specific attention. Among these, there is the confirmation of higher survival rates of lymphomas as compared to carcinomas of the stomach and, perhaps, of the oral cavity (Mittal et al., 1983); among small bowel cancers, the proportion of infiltrating carcinoids is similar to that of adenocarcinomas and the survival rate for these two histotypes is comparable (approximately 35% at 5 years); overall, survival from small bowel cancers (29% at 5 years) is only slightly lower than for the globality of colon (34%) or rectal (37%) cancers. In relation to large bowel cancers, survival was, as expected, higher for carcinomas arising on polyps, but there is, to our knowledge, no simple explanation for the larger proportion of squamous and transitional cell cancers in females (although this has not important prognostic implications).

The elevated proportion of undefined histological types for liver and pancreatic cancers underlines the difficulties of precise case ascertainment and classification for these neoplasms (Doll & Peto, 1981), even in a highly integrated and monitored population-based scheme.

The higher frequency of cholangio- and hepatocellular carcinomas combined in females than in males probably reflects similarities of aetiological correlates with gallbladder cancer, although the issue is still discussed (Strom et al., 1985), Prognosis was extremely poor for all neoplasms arising from liver, gallbladder and pancreas (Propok, 1978; Levine et al., 1981; Doll & Peto, 1981; Cairns & Boyle, 1983; American Cancer Society, 1988), but there was some suggestion that intrahepatic cholangiocellular carcinomas have lower survival than gallbladder cancers.

These and several other indications emerging from careful examination of the data presented herein underline the interest of morphological analyses of digestive tract neoplasms, in terms of descriptive epidemiology, inference on aetiological correlates and prognostic implications.

The authors wish to thank Dr E. Gloor and Dr J. Costa (Institut universitaire de pathologie, Lausanne) for their most helpful advice and support, and R. Grimm for computing assistance.

The contribution of the Swiss League against cancer is gratefully acknowledged. S.F. was the recipient of an ICRETT award (No 173).

### References

AMERICAN CANCER SOCIETY. CANCER STATISTICS 1988 (1988). Ca-A Cancer. *J. Clinicians*, **38**, 21.

BLENKISOPP, W.K., STEWART-BROWN, S., BLEVOSKY, L., & KEARNEY, G. (1981). Histopathology reporting in large bowel cancer. *J. Clin. Pathol.*, **34**, 509.

CAIRNS, J. & BOYLE, P. (1983). Cancer chemotherapy. *Science*, **220**, 252.

CHÉRIE-CHALLINE, L., POTTIER, D. & GIGNOUX, M. (1988). Epidémiologie descriptive du cancer de l'oesophage dans le département du Calvados: 520 cas (1978–1982). *Gastroenterol. Clin. Biol.*, **12**, 126.

DOLL, R. & PETO, R. (1981). The causes of cancer: quantitative estimates of avoidable risks of cancer in the United States today. *J. Natl Cancer Inst.*, **66**, 1191.

FAIVRE, J., ANGLESIO, E., RAYMOND, L., SCHAFFER, P., TUYNS, A. & ZUBIRI, A. (1979*a*). Distribution géographique des cancers digestifs dans quelques régions de pays latins. *Rev. Epidém. Santé Publ.*, **27**, 499.

FAIVRE, J., KLEPPING, C., MARTIN, F., CABANNE, F., MICHIELS, R. & DUSSERRE, P. (1979*b*). Incidence des cancers digestifs dans une population bien définie. Bilan de deux années d'enregistrement dans le département de la Côte-d'Or. *Rev. Epidém. Santé Publ.*, **27**, 41.

FAIVRE, J., JUSTRABO, E., HILLON, P., MILAN, C. & KLEPPING, C. (1985). Gastric carcinoma in Côte d'Or (France). A population-based study. *Gastroenterol.*, **88**, 1874.

FAIVRE, J., BEDENNE, L., BOUTRON, M.C., MILAN, C., COLLONGES, R. & ARVEUX, P. (1989). Epidemiological evidence for distinguishing subsites of colorectal cancer. *J. Epidemiol. Comm. Hlth.*, **43**, 356.

KIMURA, W., SHIMADA, H., KURODA, A. & MORIOKA, Y. (1989). Carcinoma of the gallbladder and extrahepatic bile duct in autopsy cases of the aged, with special reference to its relationship to gallstones. *Am. J. Gastroenterol.*, **84**, 386.

KUNE, S., KUNE, G.A. & WATSON, L. (1986). The Melbourne colorectal cancer study: incidence findings by age, sex, site, migrants and religion. *Int. J. Epidemiol.*, **15**, 483.

LAVECCHIA, C. & LEVI, F. (1988). Sex differentials in Swiss cancer mortality. *Soz. Praeventivmed.*, **33**, 140.

LEVI, F. (1987). Statistics from the registry of the canton of Vaud, Switzerland, 1978–1982. In *Cancer Incidence in Five Continents*, Muir, C.S., Waterhouse, J., Mack, T., Powell, J. & Whelan, S. (eds), Vol. V. pp. 634–639. IARC Scient. Publ. 88: Lyon.

LEVI, F., MAISONNEUVE, P., FILIBERTI, R., LAVECCHIA, C. & BOYLE, P. (1989). Cancer incidence and mortality in Europe. *Soz. Praeventivmed.*, **34**, (Suppl. 2). S1.

LEVINE, D.L., CONNELLY, R.R. & DEVESA, S.S. (1981). Demographic characteristics of cancer of the pancreas: mortality, incidence and survival. *Cancer*, **47**, 1456.

MCMICHAEL, A.J. & POTTER, J.D. (1980). Reproduction, endogenous and exogenous hormones, and colon cancer: a review and hypothesis. *J. Natl Cancer Inst.*, **65**, 1201.

MCMICHAEL, A.J. & POTTER, J.D. (1983). Do intrinsic sex differences in lower alimentary tract physiology influence the sex-specific risks of bowel cancer and other biliary and intestinal disease? *Am. J. Epidemiol.*, **118**, 620.

MITTAL, B., WASSERMAN, T.H. & GRIFFITHS, R.C. (1983). Non-Hodgkin's lymphoma of the stomach. *Am. J. Gastroenterol.*, **78**, 780.

MUIR, C.S., WATERHOUSE, J.A.H., POWELL, J., MACK, T. & WHELAN, S. (1987). (eds) In *Cancer Incidence in Five Continents*. Volume IV, IARC Scient. Publ., 88: Lyon.

PETERS, R.K., PIKE, M.C., CHANG, W.W.L. & MACK, T.M. (1990). Reproductive factors and colon cancer. *Br. J. Cancer*, **61**, 741.

PETO, R., PIKE, M.C., ARMITAGE, P. & 7 others (1977). Design and analysis of randomized clinical trials requiring prolonged observation of each patient. Part II: Analysis and examples. *Br. J. Cancer*, **35**, 1.

PROPOK, P. (1978). Survival for cancer of the digestive system. End results selection. *Natl Cancer Inst., DHEW Publ.* No NIH 78-1541, Bethesda.

SERVICE CANTONAL DE RECHERCHE ET D'INFORMATION STATISTIQUES (SCRIS). *Annuaire statistique du canton de Vaud*. Lausanne, various issues.

STROM, B.L., HIBBERD, P.L., SOPER, K.A., STOLLEY, P.D. & NELSON, W.L. (1985). International variation in epidemiology of cancers of the extrahepatic biliary tract. *Cancer Res.*, **45**, 5165.

WEBER, W., TORHORST, J. & OBRECHT, J.P. (1980). Die Epidemiologie des Pankreas-karzinoms in Basel. *Schweiz. med. Wschr.*, **110**, 857.

WIDGREN, S. (1980). Le cancer du pancréas à Genève. Etude anatomopatholigque de 177 cas. *Schweiz. med. Wschr.*, **110**, 447.

WORLD HEALTH ORGANIZATION (1976). *International Classification of Diseases for Oncology*. WHO: Geneva.

YANG, P.C. & DAVIS, S. (1988). Incidence of cancer of the esophagus in the US by histologic type. *Cancer*, **61**, 612.

ZARIDZE, D.G. & FILIPCHENKO, V.V. (1990). Incidence of colo-rectal cancer in Moscow (Letter). *Int. J. Cancer*, **45**, 583.

ZUR HAUSEN, H. (1989). Papillomaviruses as carcinomaviruses. In *Advances in Viral Oncology*. Klein, G. (ed.), vol 8. Raven Press: New York, 1.